# miR-21-5p inhibitor enhances the radiosensitivity of human cervical cancer cells via blocking CPEB3-mediated CDK1/cyclin B pathway

**DOI:** 10.1016/j.bbrep.2025.102177

**Published:** 2025-07-25

**Authors:** Liang Jiao, Yuhua Gao

**Affiliations:** Cancer Hospital of China Medical University, Liaoning Cancer Hospital& Institute, Shenyang, Liaoning, 110042, China

**Keywords:** miR-21-5p, Radiotherapy, Radio-sensitivity, CDK1, Cyclin B, Cervical cancer

## Abstract

Currently, radiotherapy remains the standard treatment for human cervical cancer (CC), while approximately 30 % of patients are still non-responsive to radiotherapy, leading to radioresistance. Therefore, it is urgent to discover a novel therapeutic target/biomarker for the radiosensitivity of cervical cancer, and it will be beneficial to clinical guiding significance for individualized treatment of cervical cancer. Herein, the correlation of miR-21-5p and clinicopathological features, clinical efficacy, radiotherapy sensitivity and survival were investigated, and our results showed that miR-21-5p is positively correlated to radio-sensitivity in patients with CC. Furthermore, we found that inhibition of miR-21-5p significantly suppressed the growth of HeLa and SiHa cells, and induced early apoptosis. Meanwhile, miR-21-5p inhibitor caused an arrest cell cycle at G2/M phase. Of note, the miR-21-5p inhibitor significantly enhanced the efficacy of radiation in a dose and timing-dependent manner in human cervical cancer cells. Mechanistically, our results demonstrated that miR-21-5p inhibitior directly up-regulated CPEB3 and further suppressed CDK1/cyclin B pathway. The blockage of CDK1/cyclin B is responsible to the suppression of miR-21-5p and arresting cell cycle at G2/M. Taken together, miR-21-5p can serve as a potential predictor/biomarker for radioresistance and poor prognosis of CC patients. Our results suggested that miR-21-5p inhibitor effectively restored the radio-sensitivity of CC cells through blocking CPEB3-mediated CDK1/Cyclin B pathway.

## Highlights

• MiR-21-5p is positively correlated to radio-sensitivity in patients with cervical cancer.
• MiR-21-5p inhibitor arrested cell cycle at G2/M phase and induced cellular apoptosis.
• MiR-21-5p inhibitor enhances the radio-sensitivity of cervical cancer cells via blocking CPEB3/CDK1/Cyclin B pathway.

## Graphical abstract

**Figure:**
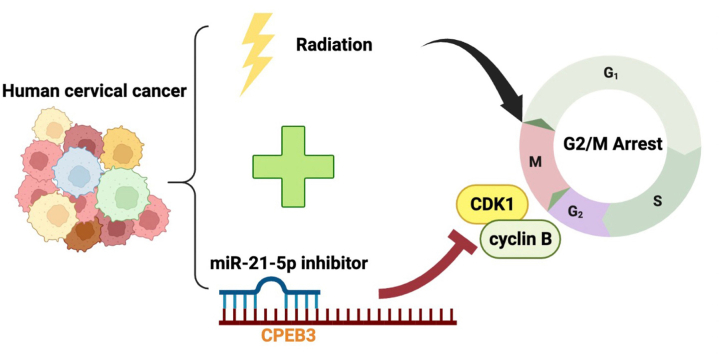

## 1 Introduction

Currently, cervical cancer (CC) is ranked as the 4th cancer in women worldwide [1]. Over 500,000 new patients were diagnosed, and it estimated 260,000 deaths per year [2]. Tumor invasion and distant metastasis are the main causes for treatment failure in CC patients. Although there are relatively reliable screening methods for precancerous lesions, most of the patients in China are not diagnosed at an early stage and cannot be treated with surgery [3].

Radiotherapy remains the standard treatment for human cercial cancer. Unfortunately, 30 % of CC patients are still less therapeutic response to radiotherapy, leading to disease progression and recurrence [4,5]. Therefore, it is urgent to discover the novel therapeutic target/biomarker for the radiosensitivity of CC, and it will be benefit to clinical guiding significance for individualized treatment of cervical cancer.

MicroRNA (microRNA, miRNA) is a class of endogenous small molecule non-coding RNA, which is an important post-transcriptional regulator and participates in almost all activities in the multiple process of tumor growth and resistance [[6], [7], [8]]. Of note, researchers found that miRNAs could impact on the chemo-sensitivity of CC cells through regulating downstream target genes, suggesting that miRNAs may be potential targets for CC diagnosis, treatment, and poor prognosis [9]. Jia et al. [10] detected the expression of circulating miR-21-5p in the serum of 20 CC patients by high-through sequencing and discovered that the AUC value of miR-21-5p was significantly higher than that of squamous cell carcinoma antigen and CA125 [11]. The evidences shown that miR-21-5p can be used as a tumor marker for the early non-invasive diagnosis of cervical cancer. To date, the relationship between miR-21-5p expression and radiosensitivity and clinicopathological characteristics in CC patients is still known.

In this study, we aim to investigate the correlation of miR-21-5p expression and radiosensitivity of CC patients, and to evaluate its potential as a biomarker/therapeutic target for human CC.

## 2 Materials and methods

### 2.1 Cell lines and cell culture

The mammary epithelial cell line HeLa and SiHa were obtained from the Cell Bank of the Chinese Academy of Sciences (Shanghai, China). The cells were cultured in high-glucose DMEM (Hyclone, UT, USA) contained 10 % FBS (Gibco, Waltham, MA, USA). at 37 °C in a 5 % CO_2_.

### 2.2 Patients and tissue samples

Our study was reviewed and approved by the ethics committee of the Liaoning Cancer Hospital (Ethics Review Approval No. 20181206). The patients or the immediate family members signed written informed consents. Totally, 111 female participants with stage IIB-IV cervical cancer accepted concurrent chemoradiotherapy in Liaoning Cancer Hospital from October 2017 to February 2019 was enrolled.

### 2.3 miRNA extract and RT-PCR

miRNA extract was perform by miRNeasy Tissue/Cells Advanced Micro Kit (QIAGEN, #217684). miR-21-5p expression level was measured by real-time quantitative PCR (RT-PCR). The miR-21-5p forward primer sequence (5′–3′) is 5′- ACG TGT TAG CTT ATC AGA CTG-3’. The relationship between clinicopathological features, clinical efficacy, radiotherapy sensitivity and survival were statistical analyzed.

### 2.4 Cell growth assay

Cell proliferation was measured by xCELLigence Real-Time Cell Analyzer (RTCA)-MP system (ACEA Biosciences, Inc., San Diego, CA, USA). 1 × 10^5^ cells/ml in 100 μl complete medium was seeded into E−16 plates. This is included a biocompatible microelectrode array. Cell viability was recorded in every 5 min and shown as the cell index. Finally, cell index was normalized to the time point of agent treatment.

### 2.5 miRNA transfection

According to the manufacturer's instructions, plasmids were transfected using Lipofectamine 2000 (Invitrogen, USA). The inhibitors for miR-21-5p (5′-UCAACAUCAGUCUGAUAAGCUA-3′), non-specific control (5′-UCUACUCUUUCUAGGAGGUUGUGA-3′); wild-type and mutant type dual-luciferase reporter plasmids were purchased from Genomeditech Co. (Shanghai, China).

### 2.6 Clonogenic assay

2000 cells/well were seeded into a 6-well plate. After overnight miR-21-5p inhibitor or control were tranfected for additional 48 h. Fresh medium was changed for every 3–4 days. 14 days later, the colonies were stained by crystal violet and further quantified.

### 2.7 Apoptosis analysis

As previously described [12], 5 × 10^5^ cells was harvested and stained for 10 min with 5 μl Annexin V-fluorescein isothiocyanate (FITC) and/or 5 μl propidium iodide (PI) in 500 μl buffer. Then, cells were rinsed twice with cold PBS. Apoptotic cells was analyzed by flow cytometry (BD Accuri™ C6, BD Biosciences, NJ, USA).

### 2.8 Western blotting analysis

The information of primary antibodies as follow CDK1 (CST #18048), cyclin B (CST # #12231), β-actin (CST #3700), CPEB3 (Invitrogen #12669-1-AP). The secondary antibodies are goat anti-mouse IgG-HRP (Invitrogen #31430; dilution = 1:10,000) or goat anti-rabbit IgG-HRP (Invitrogen #31460; dilution = 1:15,000) against the primary antibodies. Bio-Rad GelDoc XR + system (Bio-Rad Laboratories, Inc., USA) were used to immunoblot analysis.

### 2.9 Statistical analysis

Data were shown as mean ± SD. The Student's t-test (two-tailed) was used for two group comparisons. One-way ANOVA was used to comparisons between groups of more than two unpaired values.The SPSS software (version 21, IBM Corp., USA) was used for statistical analysis. *p* < 0.05 means statistically significant.

## 3 Results

### 3.1 The correlation of miR-21-5p and radio-sensitivity in human cervical cancer

**Fig. 1:**
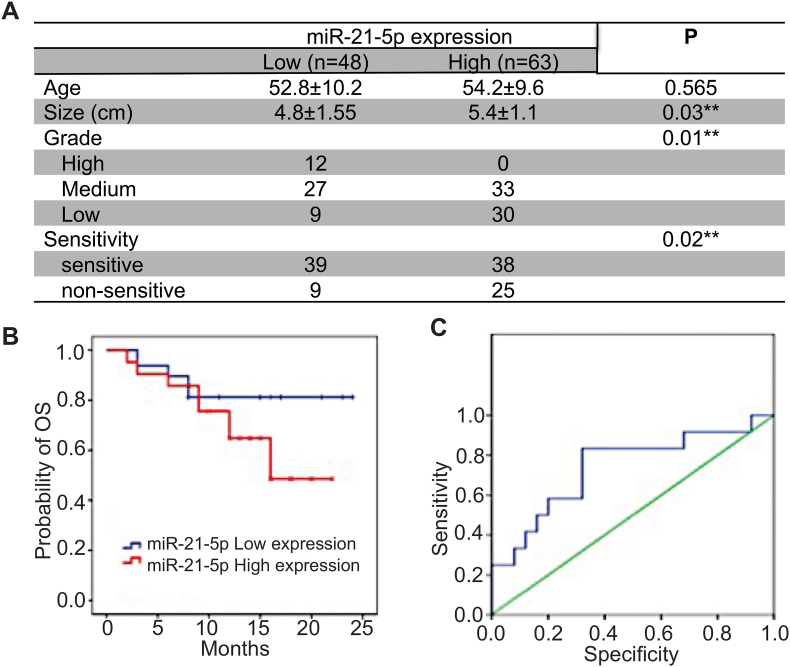
The correlation of miR-21-5p and radio-sensitivity in human cervical cancer. (A) The relationship between miR-21-5p and clinicpathological feature, radiosensitivity of patients with advanced cervical cancer; (B) Correlation of miR-21-5p expression and the overall survival (OS) of patients with advanced cervical cancer was analyzed by Kaplan-Meier curve and log rank test (p = 0.022); (C) Kapla-Meier analysis of the relationship between miR-21-5p expression and PFS in patients with advanced cervical cancer.∗p < 0.05; ∗∗p < 0.01.

Totally, 111 female CC patients with stage IIB-IV cervical cancer were enrolled, including radio-sensitive group (75 cases) and radio-resistance group (36 cases). The tumor size (diameter, cm) of radiotherapy resistance group was markerdly larger than that in the sensitive group (p < 0.01). There are no significant differences in other general clinical data between the radio-sensitive and radio-resistance groups (p > 0.05). The overexpression of miR-21-5p was positively correlated to the tumor size and radio-sensitivity (p < 0.05). The OS (overall survival) of CC patients with high expression of miR-21-5p were lower than that of low-miR-21-5p in CC patients (Log-rank test, p < 0.05). Multivariate Cox regression analysis showed that the factors influencing PFS included miR-21-5p level and tumor size (p < 0.05). The curve showed that the sensitivity of miR-21-5p in the diagnosis of CC radio-sensitivity was 83.3 %, and the specificity was 68.0 %. Taken together, miR-21-5p is closely associated with radio-sensitivity in CC patients and can be used as a promising indicator for evaluating prognosis and selecting individualized treatment (see Fig. 1).

### 3.2 The effect of miR-21-5p inhibitor on the growth of human cervical cancer cells

**Fig. 2:**
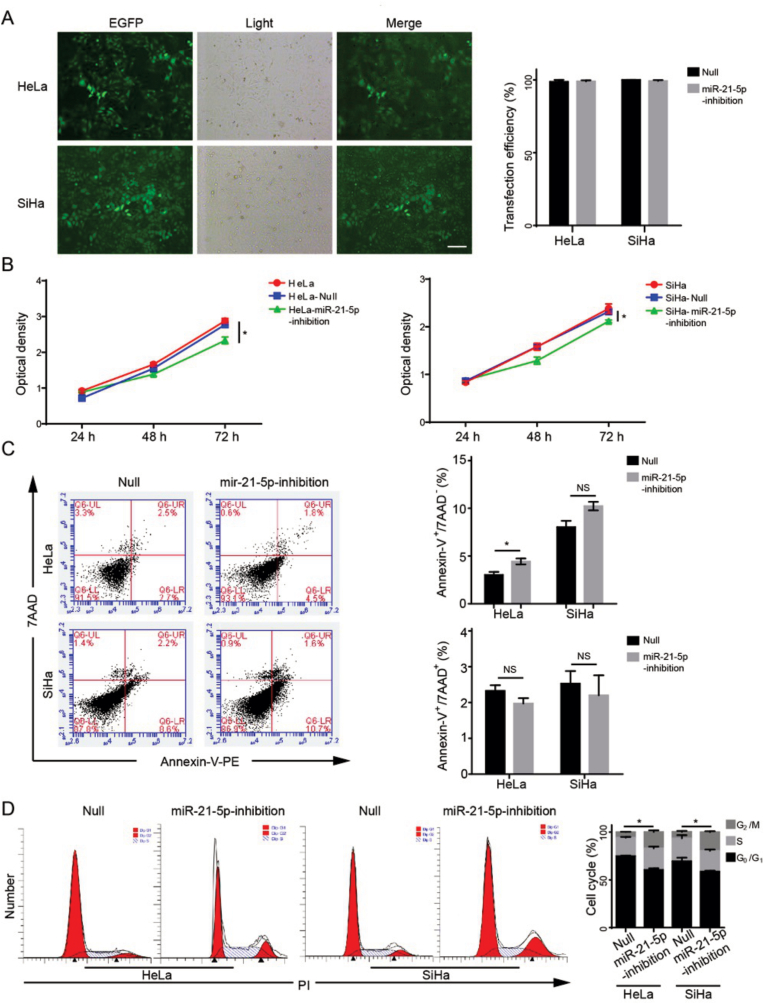
The effect of miR-21-5p inhibitor on the growth of human cervical cancer cells. (A) Transfection of miR-21-5p inhibitor into HeLa and SiHa cells, the transfection efficiency was determined, scale bar = 20μm; (B) The effect of miR-21-5p on the growth of HeLa and SiHa cells. After transfection of miR-21-5p or null control for 24, 48 and 82 h, the cell proliferation was determined by using xCELLigence Real-Time Cell Analyzer; (C) Effect of miR-21-5p inhibitor on inducing cellular apoptosis. After transfection with miR-21-5p inhibitor for 72 h, the early and late apoptotic cells were measured by Annexin V/7-AAD staining assay; (D) Effect of miR-21-5p on the distribution of cell cycle. After treatment of miR-21-5p inhibitor, the alteration of cell cycle was determined by flow cytometry, ∗p < 0.05.

To test miR-21-5p on the growth of human CC cells, the miR-21-5p inhibitor and control were constructed and transfected. As shown in Fig. 2A, the transfection efficiency is nearly 100 %, it means that miR-21-5p inhibitor could be effective transfected into the cells. After that, the effect of miR-21-5p inhibitor on the growth of HeLa and SiHa cells was determined by xCELLigence Real-Time Cell Analyzer. As shown in Fig. 2B, miR-21-5p inhibitor significantly reduced the proliferation of HeLa and SiHa cells, as compare to the negative control group (p < 0.05). Given that the inhibitory effect on cell growth, the effect of miR-21-5p inhibitor on inducing cellular apoptosis was measured by flow cytometry. miR-21-5p inhibitor significantly increased the early apoptotic cell (Annexin V+/7-AAD-), but did not significantly affect the late apoptotic cells (Annexin V+/7-AAD+, Fig. 2C). It suggested that the miR-21-5p inhibitor only caused an increase of early apoptotic cells. In addition to cellular apoptosis analysis, the alteration of cell cycle by miR-21-5p inhibitor were also determined by flow cytometry. As seen in Fig. 2D, the percentage of G2/M cells were significantly increased by treatment of miR-21-5p inhibitor, and it suggested that suppression of miR-21-5p lead to block cell cycle at G2/M phase.

### 3.3 miR-21-5p inhibition enhances the efficacy of radiotherapy in human cervical cancer

**Fig. 3:**
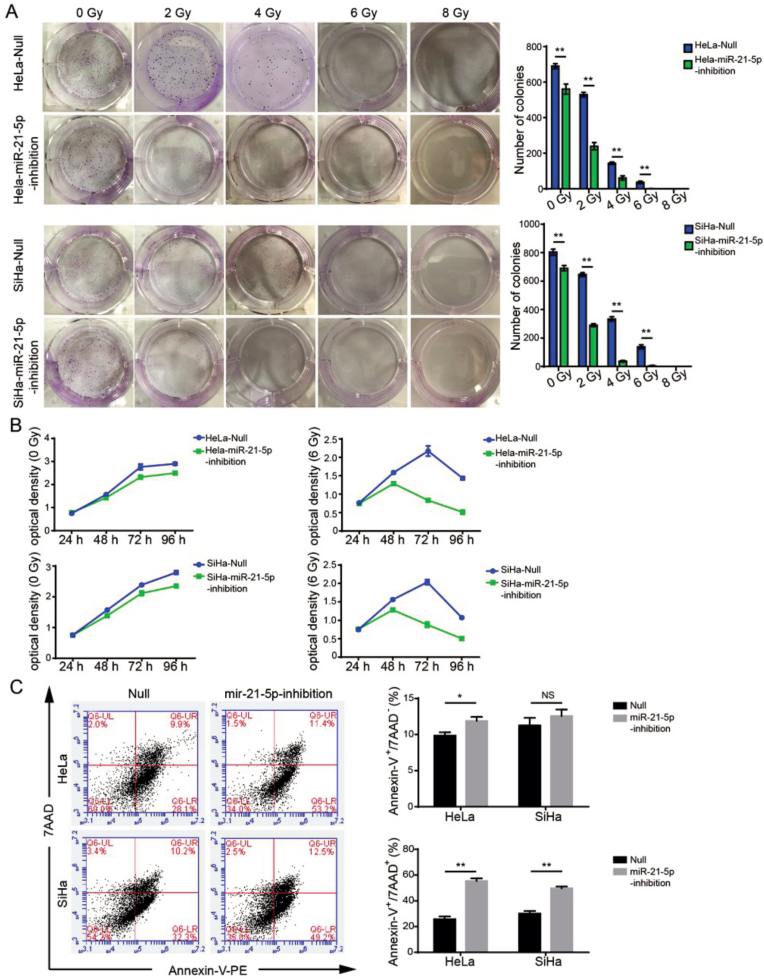
miR-21-5p inhibition enhances the efficacy of radiotherapy in human cervical cancer. (A) Colony formation was used to determine the radio-sensitive effect of miR-21-5p in human cervical cancer cells; (B)miR-21-5p inhibitor sensitize the efficacy of radiation in human cervical cancer cells. HeLa or SiHa cells were treated by radiation (0 or 6 Gy) with/without miR-21-5p inhibitor for different time-point, the cell growth was determined by using xCELLigence Real-Time Cell Analyzer; (C) miR-21-5p inhibitor synergistically enhanced the radiation in inducing cellular apoptosis in human cervical cancer cells. The cells were treated by radiation (0 or 6 Gy) with/without miR-21-5p inhibitor for 72 h, the early and late apoptotic cells were detected by Annexin V/7-AAD staining assay. ∗p < 0.05.

To confirm the miR-21-5p inhibitor enhance radiotherapy in human CC model, the synergistic effect of miR-21-5p inhibitor and radiation on the cell growth was investigated by using colony formation assay and xCELLigence Real-Time Cell Analyzer. As seen in Fig. 3A, the radiation alone could suppress the growth of HeLa and SiHa cells in a dose-dependent manner. Moreover, we observed that miR-21-5p inhibitor significantly enhanced the cytotoxic effect of radiation on colony formation of HeLa and SiHa cells. Especially, under the dose of 6 Gy, the combination of miR-21-5p inhibitor and radiation generate a greater killing effect on the cell growth than the radiation alone group, and almost eradicate all the viable cells. In addition to colony formation assay, the synergistic effect of miR-21-5p inhibitor and radiation was validated by xCELLigence Real-Time Cell Analyzer. There is a markedly difference between miR-21-5p inhibitor alone and in combination with radiation (6 Gy, Fig. 3B). miR-21-5p inhibitor significantly improved the efficacy of radiation. After 72 h’ treatment, the survival cells were significantly reduced in miR-21-5p inhibitor combined with 6Gy radiation group (p < 0.01). This result is consistent to colony formation data.

We next detected the synergistic effect of miR-21-5p inhibitor and radiation on inducing cellular apoptosis. After the cells were treated by miR-21-5p inhibitor with/without radiation (6Gy), early and late apoptotic cells were measured by Annexin V/7-AAD staining analysis. As seen in Fig. 3C, radiation (6 Gy) alone resulted in an increase of 10 % early apoptosis and 20 % late apoptosis, while the combination of miR-21-5p inhibitor significantly increased the percentage of early and late apoptotic cells to ∼15 % and ∼50 %, respectively. Thus, miR-21-5p inhibitor evidently enhanced the radio-sensitivity of human CC cells.

### 3.4 miR-21-5p inhibitor directly targeting CPEB3-mediated regulation of CDK1/cyclin B

**Fig. 4:**
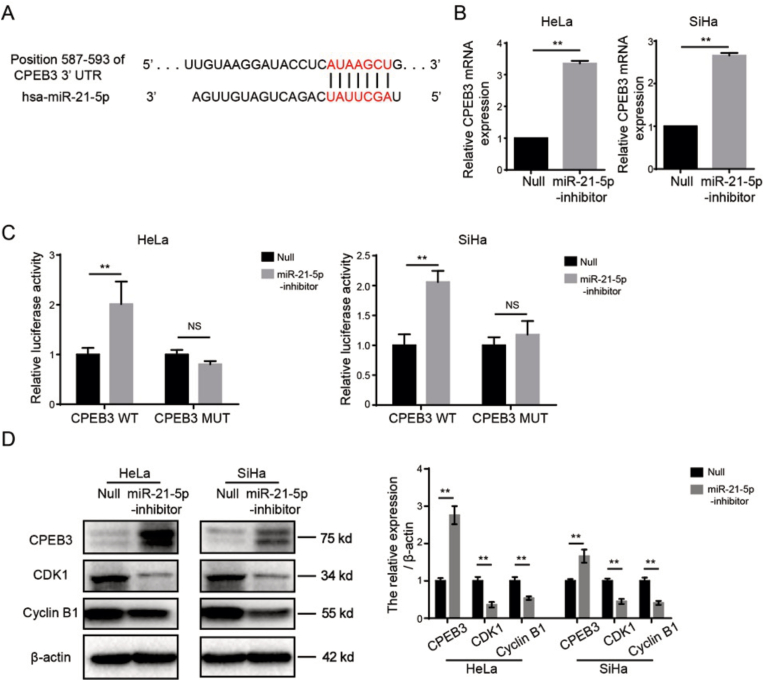
miR-21-5p inhibitor directly targeting CPEB3-mediated suppression of CDK1/cyclin B. (A) Bioinformatics analysis revealed the predicted binding sites between miR-21-5p and CPEB3; (B) Effect of miR-21-5p on mRNA expression level of CPEB3 in human cervical cancer cells. HeLa or SiHa cells were transfected with miR-21-5p for 48 h, the mRNA expression level of CPEB3 was determined by RT-PCR; (C) Luciferase reporter assay was performed to measure the binding activity and key binding sites between CPEB3 and miR-21-5p; (D) Effect of miR-21-5p on the expression of CPEB3-mediated CDK1/cyclin B. HeLa or SiHa cells were treated by miR-21-5p inhibitor for 48 h, the expression of CPEB3, CDK1, Cyclin B and β-actin were determined by immunoblot analysis; ∗∗p < 0.01.

To clarify the mechanism of miR-21-5p on enhancing radio-sensitivity, bioinformatics analysis was performed by using TargetScanHuman website (https://www.targetscan.org/vert_80/). As shown in Fig. 4A, Bioinformatics analysis predicted that CPEB3 contains conserved target site of miR-21-5p, and the potential binding sites between miR-21-5p and CPEB3 was shown. It suggested that CPEB3 can be served as a candidate target of miR-21-5p by using bioinformatics analysis. To verify this finding, HeLa and SiHa cells were treated by miR-21-5p inhibitor, and the mRNA level of CPEB3 was quantified by RT-PCR. As seen in Fig. 4B, inhibition of miR-21-5p significantly up-regulated the mRNA level of CPEB3. This result suggested that the correlations of miR-21-5p and CPEB3, and CPEB3 can be the downstream target of miR-21-5p.

To further analyze the directly interaction between miR-21-5p and CPEB3, wild-type and coorespoding mutant CPEB3 3′UTR luciferase reporter vector were designed and co-transfected in combination with/without miR-21-5p inhibitor into HeLa and SiHa cells, respectively. Interestingly, miR-21-5p inhibitor resulted in a significantly increase of luciferase activity of CPEB3-wt, while it showed no effect on the luciferase activity of CPEB3-mut (Fig. 4C). It means that there is directly interaction of miR-21-5p and CPEB3.

Next, miR-21-5p inhibitor significantly induced CPEB3 expression in HeLa and SiHa cells which were determined by immunoblot analysis (Fig. 4D). This result further validated that CPEB3 was the downstream target of miR-21-5p in human CC cells. In addition to the CPEB3, the alteration of downstream target/pathway were further investigated. We found that CDK1 and cyclin B were significantly reduced by miR-21-5p inhibitor. Moreover, the CDK1/cyclin B known as the key pathway which can be regulated by CPEB3. Of note, the blockage of CDK1/cyclin B resulted in G2/M phase arrest, this result is consistent to our previous data. Thus, our findings suggested that miR-21-5p inhibitor directly induced CPEB3 expression, suppressed CDK1/cyclin B pathway, and finally arrested cell cycle at G2/M phase.

## 4 Discussion

miRNAs has been investigated in human cervical cancer, including miR-155 [13], miR-126 [14], miR-425-5p [15], etc. These miRNA can manipulate the expression of different target genes, and impact on cell proliferation, apoptosis, invasion, metastasis and angiogenesis. These alterations are associated with clinical stage, vascular invasion, HPV infection, histological grade, etc [[16], [17], [18], [19], [20]]. Recently, miR-21-5p is overexpression in tumor tissue of patients with advanced CC, further study demonstrated that miR-21-5p altered multiple phenotype of tumor cells, including proliferation, invasion, and metastasis by regulating downstream target genes PTEN, Akt, and HIF-1α, promoting tumor progression [21,22]. In addition to miR-21-5p, miR-21-3p inhibitor exerted myocardial protective effects through altering macrophage polarization state and reducing excessive mitophagy [23]. Other studies demonstrated that miR-21 promotes STAT3-dependent gastric cancer progression [24]. Moreover, miR-21 inhibition can be served as a promising strategy to overcome drug resistance in cancer [25].

Herein, our results showed that high-level expression of miR-21-5p was positively related to the large diameter, poor differentiation of the tumor. Moreover, miR-21-5p overexpression could be an independent risk factor for poor prognosis in patients with advanced CC. Of note, increasingly evidence indicated that miRNAs could affect the radiosensitivity of tumor cells, and may be used in clinical predicator for radioresistance and sensitization targets. For example, studies found that miR-196a can be used as a predictor and candidate therapeutic target of cisplatin resistance in head and neck cancer [26]; miR-185 significantly reduced the tumor growth of renal cancer under ionizing radiation and improve the survival of patients with renal cancer [27]. We observed that expression level of miR-21-5p in the radioresistant group was markedly higher than that in the radiosensitive group. Overexpression of miR-21-5p is positively correlated to sensitivity of radiation in advanced cervical cancer.

Interestingly, targeting miRNAs could effectively overcome drug resistance. For example, miR-21 inhibitor (miR-21i) in combination with chemotherapy agents into 5FU-resistant colorectal cancer HCT-116 cells (HCT116/5-FU) significantly suppressed proliferation of 5-FU-resistant colon cancer cells. miR-21 inhibitor downregulates miR-21 expression in drug-resistant HCT116/5-FU cells, which lead to arrest cell cycle, suppress proliferation, induce apoptosis, and significantly reduces tumor growth in MC38 mice model [28]. We observed that miR-21-5p inhibitor alone cause the cell cycle arrest at G2/M. Moreover, it reported the blockage of G2/M cell cycle checkpoint could enhance the efficacy of radiotherapy [29]. According to these findings, it is possible that miR-21-5p inhibitor overcomes the radioresistance in human cervical cancer model. Radiosensitizers can be a class of promising agents that enhance injury to tumor cells by accelerating DNA damage and inducing cellular apoptosis. The combination of miR-21-5p inhibitor and radiation showed a synergistic effect on blockage of cycle arrest G2/M phase and induction of cellular apoptosis.

Based on bioinformatics analysis, CPEB3 was identified as the directly downstream target for miR-21-5p. CPEB3 belongs to a member of the cytoplasmic polyadenylation element-binding family. It can regulate the translation of mRNA and plays key roles in regulating multiple cellular functions [30]. Of note, CPEB3 participate the regulation of cell cycle, especially G2/M via CDK1/cyclin B pathway. CPEB3 inhibition led to cell cycle arrested at S and G2/M phases [31]. The CDK1 is known as the only cell cycle CDK kinase which is necessary to successful completion of M-phase in human cells [32]. During G2/M phase to phosphorylate and activate substrates, CDK1/cyclin B relocates to mitochondria, it is known as enhancing mitochondrial function and energy production and driving energy sensitive G2/M transition [33]. In our study, miR-21-5p inhibitor markedly blocked CDK1/cyclin B signaling pathway, this alteration is consistent to blocking cell cycle at G2/M.

Taken together, our findings demonstrated that high-level expression of miR-21-5p in the tumor tissue of CC patients is positively correlated to radio-sensitivity and poor prognosis. Furthermore, our results showed that miR-21-5p inhibitor effectively enhanced the radio-sensitivity of CC cells through blocking CBEP-mediated CDK1/Cyclin B pathway and inducing cellular apoptosis. Therefore, miR-21-5p can be served as a potential predictor/biomarker for poor prognosis of cervical cancer patients.

## CRediT authorship contribution statement

**Liang Jiao:** Writing – review & editing, Writing – original draft, Visualization, Validation, Investigation, Funding acquisition, Data curation, Conceptualization. **Yuhua Gao:** Writing – review & editing, Investigation, Funding acquisition, Conceptualization.

## Ethical approval

This study was approved by the ethics committee of the Liaoning Cancer Hospital (Ethics Review Approval No. 20181206). The patients or the immediate family members signed written informed consents.

## Funding information

This work was supported by Key Project of the 10.13039/501100005047Natural Science Foundation of Liaoning Province (No. 20180530013).

## Declaration of competing interest

The authors declare that they have no competing interests.

## Data Availability

Data will be made available on request.
