# Supplementary material for: miR-21-5p inhibitor enhances the radiosensitivity of human cervical cancer cells via blocking CPEB3-mediated CDK1/cyclin B pathway
Source: Biochem Biophys Rep. 2025 Jul 25;43:102177. doi: 10.1016/j.bbrep.2025.102177 (PMC12311963; doi:10.1016/j.bbrep.2025.102177)
Supplement: Multimedia component 1 [file mmc1.pptx]

## Slide 1
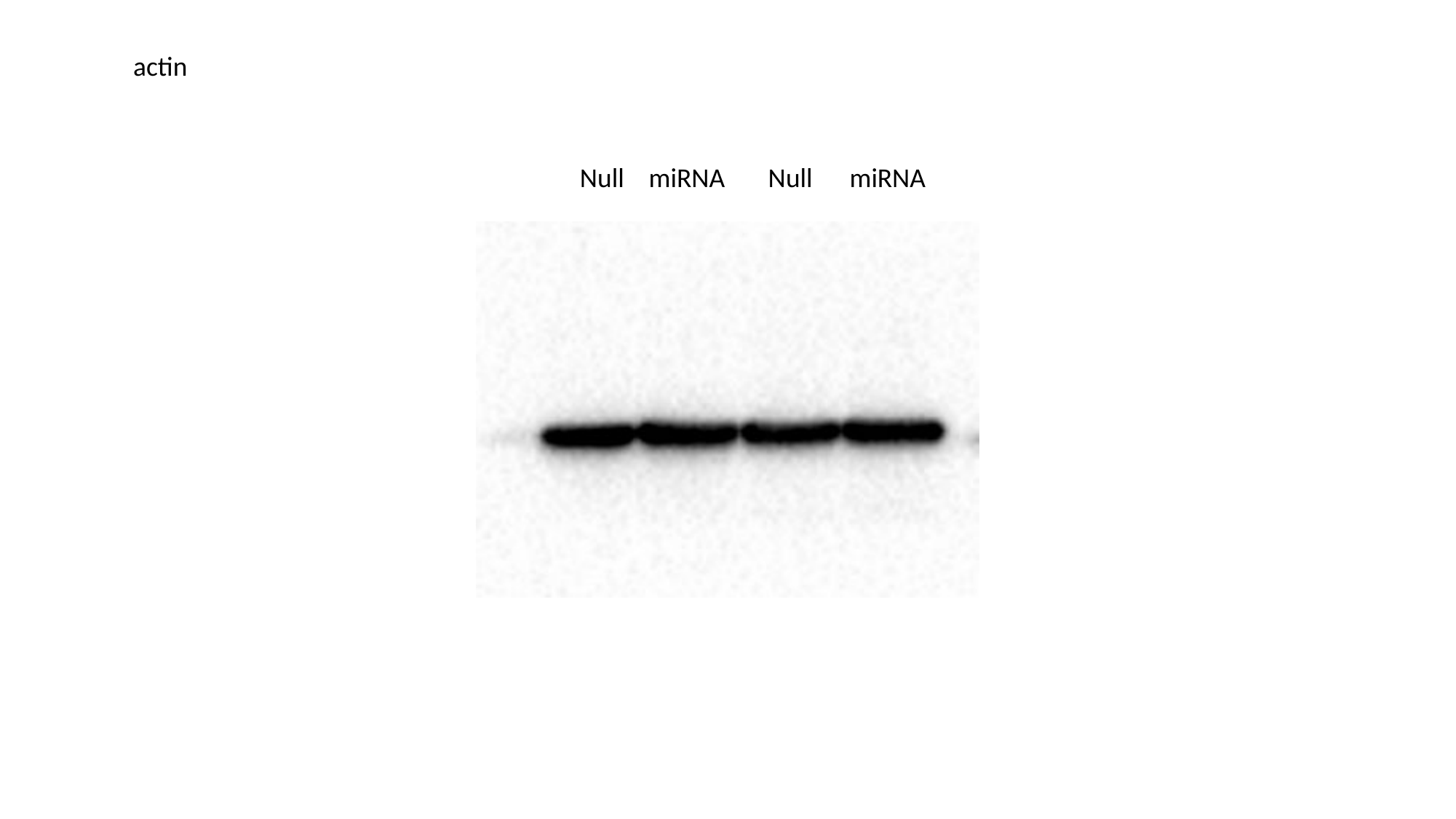

actin
Null miRNA Null miRNA

## Slide 2
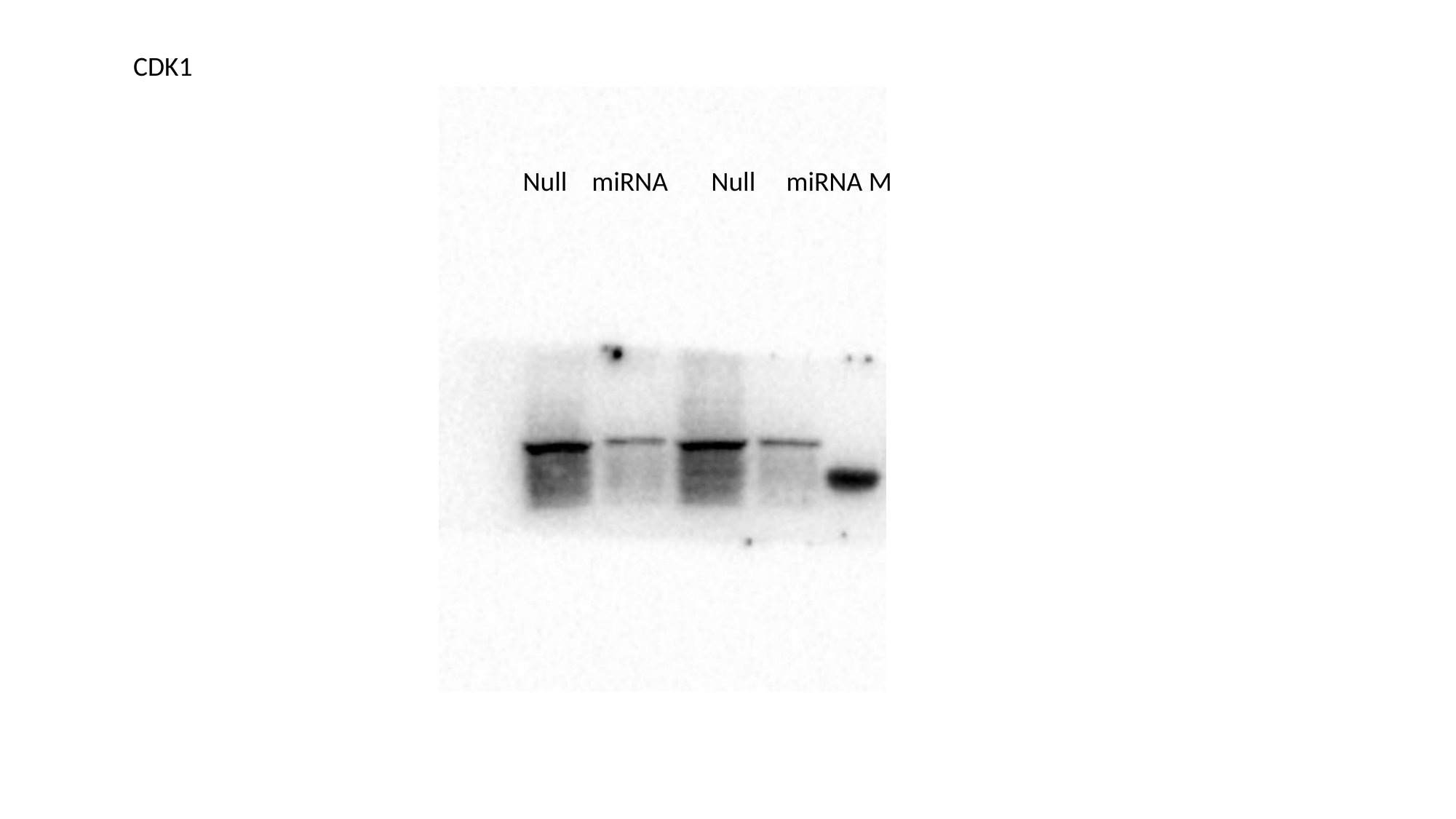

CDK1
Null miRNA Null miRNA M

## Slide 3
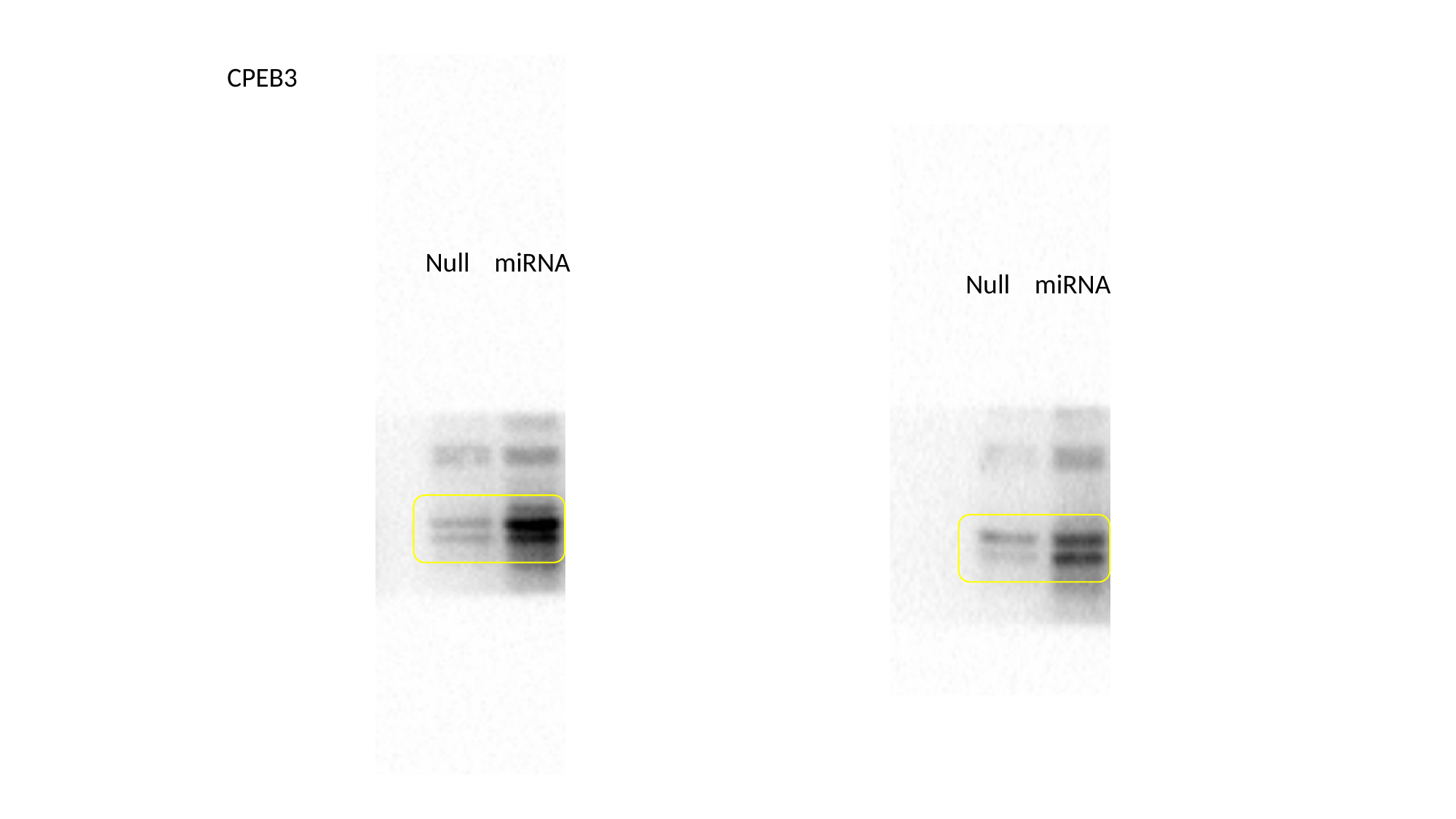

CPEB3
Null miRNA
Null miRNA

## Slide 4
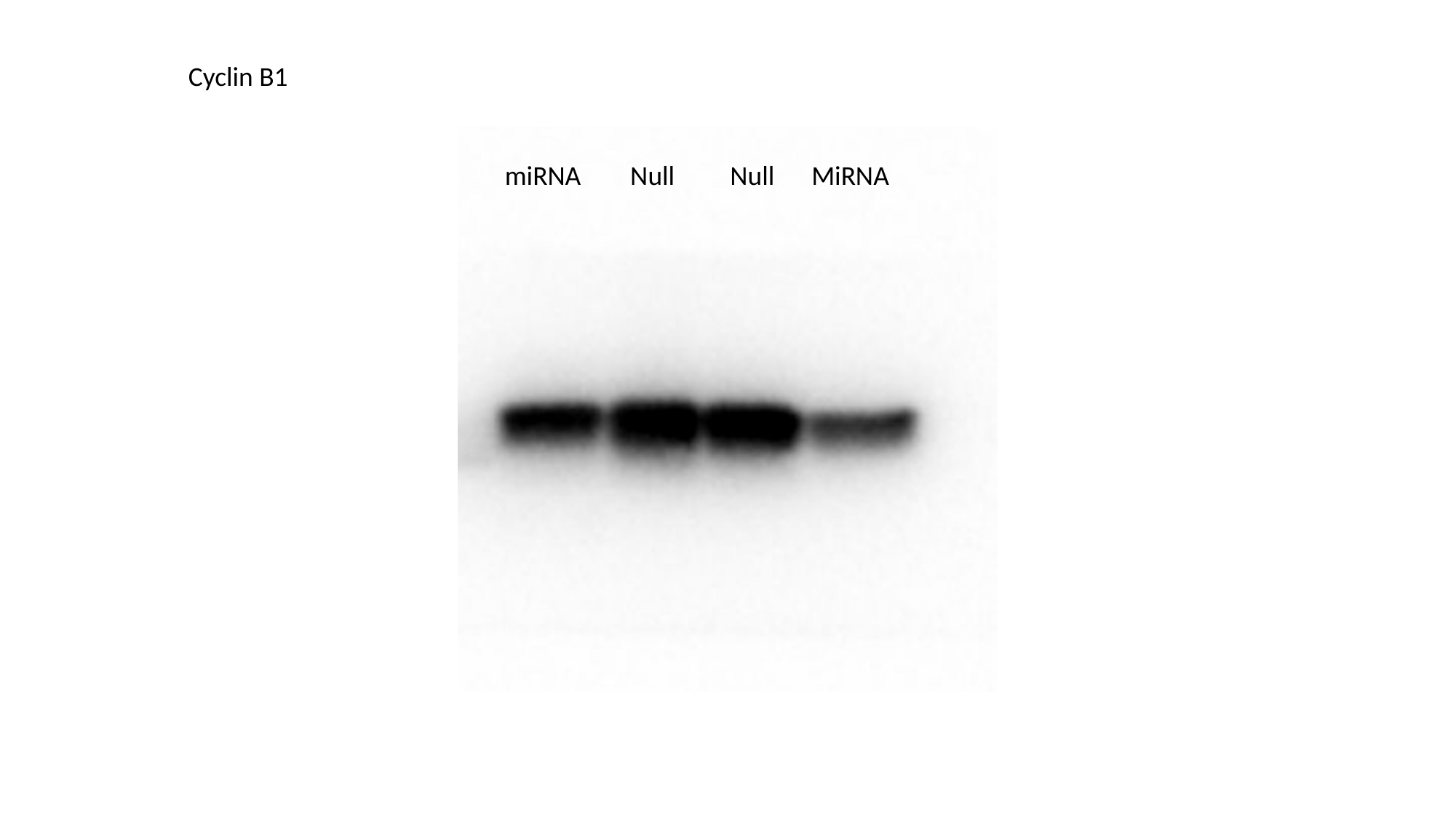

Cyclin B1
miRNA Null Null MiRNA
